# Prenatal diagnosis of Pallister‐Killian syndrome and literature review

**DOI:** 10.1111/jcmm.16853

**Published:** 2021-08-18

**Authors:** Xiaoqing Wu, Xiaorui Xie, Linjuan Su, Na Lin, Bin Liang, Nan Guo, Qingquan Chen, Liangpu Xu, Hailong Huang

**Affiliations:** ^1^ Fujian Provincial Key Laboratory for Prenatal Diagnosis and Birth Defect Medical Genetic Diagnosis and Therapy Center of Fujian Provincial Maternity and Child Hospital Affiliated Hospital of Fujian Medical University Fuzhou China; ^2^ Department of Laboratory Medicine Fujian Medical University Fuzhou China

**Keywords:** copy number variants sequencing, fluorescence in situ hybridization, nucleotide polymorphism array, Pallister‐Killian syndrome, prenatal diagnosis, ultrasound manifestation

## Abstract

Pallister‐Killian syndrome (PKS) is a rare sporadic genetic disorder usually caused by mosaicism of an extra isochromosome of 12p (i(12p)). This retrospective study analysed the prenatal ultrasound manifestations and molecular and cytogenetic results of five PKS foetuses. Samples of amniotic fluid and/or cord blood, skin biopsy and placenta were collected. Conventional karyotyping and single nucleotide polymorphism array (SNP array) were performed on all the amniotic fluid or cord blood samples. Copy number variants sequencing (CNV‐seq) and fluorescence in situ hybridization (FISH) were also used for the validation for one foetus. All the five foetuses were from pregnancies with advanced parental age. Two foetuses involved structural abnormalities and one foetus had only soft markers, all of which included increased nuchal translucency. The rest two foetuses had normal ultrasounds in the second trimester, which has rarely been reported before. The karyotype revealed typical i(12p) in four cases and a small supernumerary marker chromosome consisting of 12p and 20p in the remaining one case. The proportion of cells with i(12p) ranged from 0 to 100% in cultural cells, while SNP array results suggested 2−4 copies of 12p. For one foetus, metaphase FISH showed normal results, but the interphase FISH suggested cell lines with two, three and four copies of 12p in the amniotic fluid. Advanced parental age may be an important risk factor for PKS, and there were no typical ultrasound manifestations related to PKS. A combination of karyotype analysis and molecular diagnosis is an effective method for the diagnosis of PKS.

## 1 INTRODUCTION

Pallister‐Killian syndrome (PKS) (OMIM:#601803), also known as 12p tetraploid syndrome or 12p isobrachial (i(12p)) chromatid syndrome, was initially described by Pallister et al. in 1977^1^ and estimated to affect 1 in 20,000 live births.^2^ With the advance of molecular diagnostics, the incidence was re‐evaluated as 5.1 per 1,000,000 live births.^3^ Generally, it is cytogenetically characterized by tetrasomy of 12p through mosaic supernumerary isochromosomes 12p. The clinical features are highly variable, involving a variety of phenotypes associated with multiple systems, such as craniofacial dysmorphism characterized as bitemporal alopecia and palpebral fissures, skin pigmentary anomalies, variable developmental and neurodevelopmental delays, epilepsy, congenital diaphragmatic hernia, congenital heart defects, gastrointestinal malformations and genitourinary malformations.^3^, ^4^, ^5^, ^6^, ^7^

In prenatal settings, the diagnosis of PKS is challenging. It was first diagnosed prenatally in 1985 via amniocytes as a tetrasomy of 12p. Since then, no more than 100 prenatal cases have been published.^4^ The i(12p) cells were generally tissue‐limited, and the percentages of cells with i(12p) differed significantly between different tissues in the same individual or between cultured and uncultured prenatal specimens.^2^ During amniocyte sub‐culturing, the supernumerary marker of i(12p) may decrease rapidly. Furthermore, there were no specific ultrasound abnormalities related to PKS; thus, the detection is usually incidental in prenatal settings. The clinical indicators that have been reported in foetus with PKS were diverse, including advanced maternal age,^3^, ^8^, ^9^ increased nuchal translucency (NT), thickened nuchal fold, increased prenasal thickness,^3^, ^8^, ^10^, ^11^, ^12^ nasal bone hypoplasia, polyhydramnios, diaphragmatic hernia,^13^, ^14^, ^15^ short long bones, cerebral ventriculomegaly and cardiac abnormalities.^10^ Among them, polyhydramnios, diaphragmatic hernia and rhizomelic limb shortening were the most common prenatal ultrasound abnormalities reported in PKS.^16^

With awareness about prenatal diagnosis and the development of molecular detection technology, the diagnosis of PKS prenatally has become more common. Single nucleotide polymorphism array (SNP array) can detect 10% of abnormal cells or even less if mosaicism involves the introduction of a new haplotype.^8^ Next‐generation sequencing is emerging as a viable alternative to chromosome microarray analysis, and copy number variants sequencing (CNV‐seq) was considered to have comparable detection efficiency as SNP array.^17^ Combining cytogenetic analysis of cultured cells and molecular genetic testing of uncultured specimens can improve the detection of PKS. Herein, we retrospectively analysed the clinical indications, ultrasonic manifestations, cytogenetic and molecular detection results of five foetuses with confirmed PKS and compared them with previous reports, in order to accumulate and analyse the current knowledge about the prenatal diagnosis of PKS.

## 2 MATERIALS AND METHODS

### 2.1 Patients and samples

This retrospective study reviewed the clinical features, diagnosis processes, diagnosis results and pregnancy outcomes of 5 foetuses with confirmed PKS after prenatal diagnosis in our centre. The maternal age ranged from 35 to 41 years, the paternal age ranged from 35 to 42 years and the gestational age ranged from 18 to 25 weeks. The pregnant women were referred to the Prenatal Diagnosis Center of Fujian Provincial Maternal and Children's Hospital due to the indications of advanced maternal age, accompanied with or without ultrasonic anomalies. The samples comprised amniotic fluid, cord blood, placentas or skin. The general characteristics are presented in Table 1.

**TABLE 1:** General information of the 5 foetuses of PKS

	Maternal age (y)	Paternal age (y)	Gestational age at diagnosis (w)	Ultrasonic findings	Pregnancy outcome
Foetus 1	35	35	20	Increased nuchal translucency (12w); Unilateral ventriculomegaly, short humerus and short femurs (23w)	TOP
Foetus 2	39	39	18	Increased nuchal translucency (13w)	TOP
Foetus 3	37	42	18/23	Normal	TOP
Foetus 4	38	40	23/27	Increased prenasal thickness (22w); Cleft palate, increased prenasal thickness, increased nuchal fold thickness (24w)	TOP
Foetus 5	41	38	20/25	Normal	TOP

Abbreviations: AF, amniotic fluid; CB, cord blood; TOP, termination of pregnancyw, week; y, year.

### 2.2 Conventional karyotyping

Conventional karyotyping consisted of cell culture, and G‐banded karyotyping was performed on cultured amniotic fluid and foetal cord blood according to the standard protocols in our laboratory. The karyotype was determined at a resolution of 320–500 band level.

### 2.3 SNP array

Genomic DNA was extracted from uncultured amniotic fluid, foetal cord blood, skin biopsy and placenta using a QIAGEN kit (Qiagen, Hilden, Germany) according to the manufacturer's instructions. Single nucleotide polymorphism array (SNP array) was performed on the samples of uncultured fluid and cord blood using Affymetrix CytoScan 750K array (Affymetrix Inc., Santa Clara, CA, UA), which includes 200,000 probes for single nucleotide polymorphisms and 550,000 probes for copy number variations (CNVs) distributed across the entire human genome. Chromosome Analysis Suite software (Affymetrix) and human genome version GRCh37 (hg19) were used. A resolution was generally applied: gains or losses of ≥400 kb and loss of heterozygosity (ROH) ≥10 Mb. All detected CNVs were compared with in‐house and national public CNV databases as follows: Database of Genomic Variants (DGV), Database of Chromosome Imbalance and Phenotype in Humans Using Ensemble Resources (DECIPHER), International Standards for Cytogenomic Arrays Consortium and Online Mendelian Inheritance in Man (OMIM).

### 2.4 CNV‐seq

To validate the SNP array results of foetus 4, we performed CNV‐seq on amniotic fluid and cord blood sampled from a second invasive operation, skin biopsy and placenta sampled after the termination of pregnancy. Genomic DNA was hydrolysed and DNA libraries constructed by end filling, adapter ligation and PCR amplification. The sequencing platform used was Illumina NextSeq550AR (Annoroad Gene Tech Co., Ltd., Beijing, China). Burrows‐Wheeler Aligner software was used to compare the sequencing information with the human reference genome (GRCh37, UCSC release hg19), and the detected CNVs were interrogated against publicly available databases, including Decipher, Database of Genomic Variants (DGV), 1000 genomes and Online Mendelian Inheritance in Man (OMIM), and their pathogenicity was assessed according to the guidelines outlined by the American College of Medical Genetics (ACMG)^18^ for interpretation of sequence variants.

### 2.5 FISH

FISH was performed on the amniotic fluid sampled from the second amniocentesis of foetus 4. Chromosome 12p–specific probe (12pter) and chromosome 12q–specific probe (12qter) were used for FISH studies.

## 3 RESULTS

### 3.1 Ultrasound findings

Abnormal ultrasound findings were recorded in three foetuses (foetuses 1 and 2 and foetus 4; Table 1), with increased nuchal translucency (NT) as the most frequent anomalies. Foetus 4 showed a progressive ultrasonic abnormality: increased prenasal thickness in 22 weeks of gestation, while in 24 weeks, cleft palate and increased nuchal fold thickness were found additional to increased prenasal thickness. For foetus 3 and foetus 5, no ultrasound abnormality was found in the second trimester.

### 3.2 Cytogenetic and molecular test results

**FIGURE 1:**
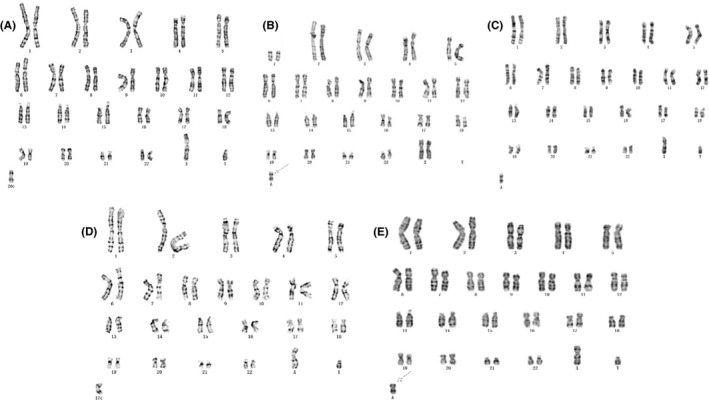
Abnormal karyotype of the 5 foetuses

The details of the results are summarized in Table 2. Foetus 1 and foetus 2 only took sample of amniotic fluid for conventional karyotyping and SNP array analysis. Foetus 1 showed a mosaicism of an extra suspected i(12p) chromosome (Figure 1A), with a mosaic proportion of 92.5%, but the SNP array results revealed three copies of 12p. Foetus 2 revealed non‐mosaicism of extra i(12p) chromosome (Figure 1B), and SNP array results suggested four copies of 12p.

**TABLE 2:** Details of the testing results for different samples

	Gestational age	Specimen	Testing results				
			Karyotype	SNP array	CNV‐seq	Metaphase FISH	Interphase FISH
Foetus 1	20^+^	AF	47,XY,+i(12p)[37]/46,XY[3] 92.5%	arr[hg19] 12p13.33q11.1(173,786‐37857931)x3	NA	NA	NA
Foetus 2	18^+^	AF	47,XY,+i(12p)	arr[hg19] 12p13.33p11.1(173,786‐34,835,641)x4	NA	NA	NA
Foetus 3	18^+^	AF	47,XY,+i(12p) [44]/46,XY[65] 40%	NA	NA	NA	NA
	22^+^	CB	47,XY,+i(12p) [3]/46,XY[82] 3.5%	arr[hg19] 12p13.33p11.1(173,786‐34,835,641)x3	NA	NA	NA
Foetus 4	23^+^	AF	46,XX	arr[hg19] 12p13.33p11.1(173,786‐34,835,641)x2‐3	NA	NA	NA
	27^+^	AF	47,XX,+i(12p) [7]/46,XY[104] 6.3%	NA	seq[GRCh37]dup(12)(p13.33p11.1)	ish 12p13q24.3(12px2,12qx2) nuc ish(12p,12q)x2[100]	nuc ish(12p)x3,(12q)x2 [19]/(12p)x4,(12q)x2 [22]/(12p,12q)x2[59]
	27^+^	CB	47,XX,+i(12p) [1]/46,XY[70] 1.4%	NA	seq[GRCh37]dup(12)(p13.33p11.1)	NA	NA
	27^+^	SB	NA	NA	seq[GRCh37]dup(12)(p13.33p11.1)	NA	NA
	27^+^	Placenta	NA	NA	seq[GRCh37]dup(12)(p13.33p11.1)	NA	NA
Foetus 5	18^+^	AF	47,XY,+mar[21]/46,XY[62] 25.3%	NA	NA	NA	NA
	25^+^	CB	47,XY,+mar[16]/46,XY[34] 32%	arr[hg19] 12p13.33p11.1(173,786‐34,759,042)x2‐3,20p13p11.1(186,793–26,129,447)x2‐3	NA	NA	NA

Abbreviations: AF, amniotic fluid; CB, cord blood; NA, not availableSB, skin biopsy.

Amniotic fluid and cord blood were collected from foetus 3 at different gestational age. The karyotypes of the both specimens were mosaicism of an extra i(12p) (Figure 1C). The proportion of abnormal cells in cord (3.5%) was significantly lower than that in amniotic fluid (40%). SNP array was performed on uncultured cord blood only, and a result of three copies of 12p was revealed.

**FIGURE 2:**
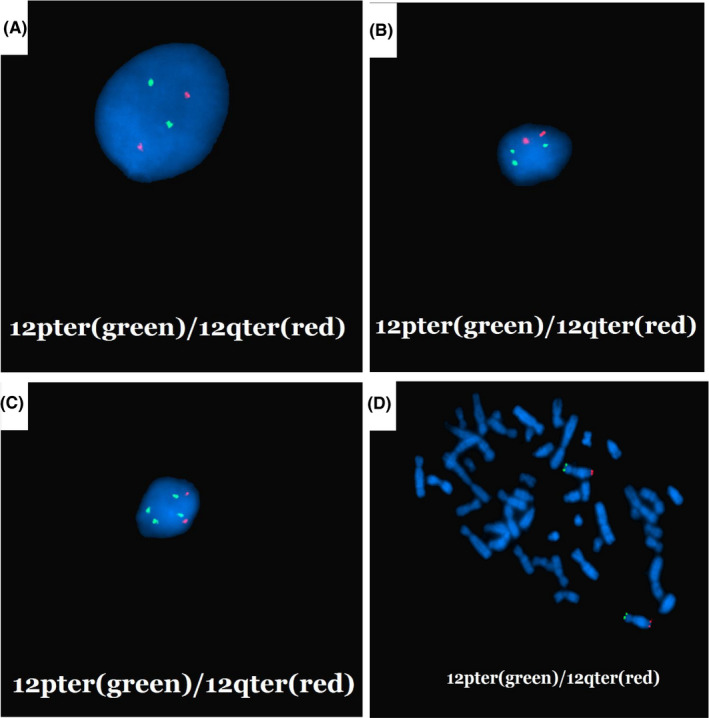
Results of FISH analyses using 12pter probe labelled green and 12qter probe labelled red on amniotic fluid of foetus 4. (A‐C) Interphase FISH. Of 100 cells analysed, 59 showed two signals of 12p, 22 showed four signals of 12p, 19 showed three signals of 12p. (D) Metaphase FISH. All of the 100 cells analysed showed two signals of 12p

For foetus 4, repeat invasive prenatal operation was performed because of the discordant results of karyotype and SNP array analysis in the first amniocentesis, that is cultured amniotic fluid showed normal karyotype, while SNP array demonstrated a result of arr[hg19] 12p13.33p11.1(173,786–34,835,641)×2‐3. For the second invasive operation, karyotype of amniotic fluid and cord blood showed very low level of mosaicism of i(12p) (Figure 1D), the CNV‐seq results of amniotic fluid, placenta and skin biopsy all suggested a duplication of 12p. For the amniotic fluid sampled in the second time, metaphase FISH showed normal result while interphase FISH suggested that the percentage of cells with (12q)×2, (12p)×3 and (12p)×4 were 59%, 19% and 22%, respectively (Figure 2).

Amniotic fluid and cord blood were also collected at gestational age of 20 and 25 weeks. The karyotype showed a mosaicism of small supernumerary marker chromosomes consisted of 20p and 12p (Figure 1E), which were confirmed by SNP array.

## 4 DISCUSSION

The PKS phenotype could be caused by a mosaicism of partial tetrasomy of 12p or a duplication of 12p due to the dosage‐sensitive genes of 12p.^19^, ^20^ In the present study, the karyotypes revealed mosaic tetrasomy of 12p in three cases, mosaic trisomy of 12p in one case and non‐mosaic tetrasomy of 12p in one case. It was reported that a minimal critical region that was located at 12p13.31 was a key player in the variable phenotypes of PKS. There were 26 potential candidate genes, among which, ING4 and CHD4 play an important role in the process of cellular transcription and reorganization of chromatin re‐modification and cell cycle and cell metabolism. However, there is no clear evidence of correlation between the genotype and phenotype of PKS,^21^ especially in prenatal settings; thus, PKS was always observed incidentally. A variety of ultrasound abnormalities have been reported in foetuses with PKS. Increased NT, which was frequently considered a statistical marker for Down syndrome, was a well‐defined soft marker for PKS^3^, ^8^, ^12^ and was always accompanied by other ultrasound abnormalities. However, it is interesting to find that in our study, two cases showed increased NT only and two cases showed normal ultrasounds in the second trimesters, which was rarely reported in previous case reports. Although severe or profound intellectual disability and never learning to walk or talk were generally associated with PKS, there were some exceptions in previous studies, that is some PKS‐affected individuals were able to learn to walk and develop some speech and even went on to receive mainstream schooling.^22^, ^23^, ^24^ This difference may be attributed to the difference in the distribution of abnormal cells in different tissues.^25^ Our data also demonstrated the importance of molecular technology in NT thickening.

Advanced maternal age (≥35 years) was observed in the majority of previously reported cases.^2^, ^8^, ^14^, ^26^ The exact mechanism behind i(12p) cell line generation has not been clearly illustrated. Several theories have been proposed, majority of which suggested maternal meiosis II non‐disjunction as a mechanism of mosaic tetrasomy of 12p^3^, ^27^ similar to the effect of the maternal age on aneuploidy pregnancies, although there is another theory of a paternal origin.^28^, ^29^ In our study, all the five cases had advanced maternal and paternal ages, which supported the association between advanced parental age and PKS. The population‐based data by Moira Blyth showed a statistically significant increase in the risk of PKS with increasing parental age^3^; however, whether maternal age, paternal age or both play a leading role was not determined.

The cytogenetic results of PKS commonly presented as a supernumerary marker of i(12p) and very rarely as 12p duplication caused by imbalanced translocation.^24^ Due to the neutralization of double dose of 12p in normal cells and quadruple dose of 12p in abnormal cells, the results of CMA and CNV‐seq generally showed 2−3 copies or 3 copies of 12p in mosaic cases. Identification of the i(12p) is challenging due to the level of diversity of the mosaicism in different tissues, the effect of phytohaemagglutinin (PHA) promoting the growth of normal cell lines over the i(12p) cells and the cytogenetic properties that resemble a chromosome 21 tetrosomy.^30^ In prenatal settings, amniotic fluid was more effective in prenatal diagnosis of PKS than cord blood.^4^ However, the supernumerary marker chromosomes are easily decreased in amniocytes during culturing,^24^ expanded cell counts are always necessary and sometimes it would be a missed diagnosis if only cultured specimen were used. The traditional cytogenetic analysis was influenced by culture, while array and sequencing technology mainly reflected dose changes, and thus, FISH was the best method to provide abnormal chromosome composition to some extent, which was well reflected in our study. The karyotype of foetus 4 was normal in first amniocentesis, while the result showed low level of mosaic duplication of 12p in the repeat amniocentesis. The mosaic level was higher in the amniotic fluid than that in the cord blood. The metaphase FISH showed negative results, while the interphase FISH revealed that there were cell lines harbouring 3 copies of 12p and 4 copies of 12p, in addition to the normal cell line in the amniotic fluid. Combined with previous reports, we suggest that regardless of the kind of sample, CMA or genomic sequencing on uncultured samples should be used for the precise diagnosis of PKS in addition to the conventional cytogenetics on cultured samples. Interphase FISH is the most effective method for clarifying the specific cell lines.

The genetic counselling of PKS is not complicated. All reported cases of PKS have been sporadic, and there are no reports of familial recurrence.^2^ Since 12p duplication from an imbalanced translocation could also result in a PKS phenotype, balanced parental chromosomal translocation should be excluded.^31^ There is no recognized correlation between the mosaic ratio and clinical severity of PKS stated in previous studies,^32^, ^33^ a possible explanation for which is that the definite mosaic ratio in each organ could not be inferred. Our data also showed no correlation between the proportion of abnormal cells and prenatal ultrasound findings. Both cytogenetic and SNP array results of foetus 2 revealed non‐mosaicism PKS; however, the ultrasound only showed NT thickening. It may be related to the gestational age of the ultrasound test as more ultrasound abnormalities appear with increasing gestational age,^3^, ^34^ similar to foetus 1 and foetus 4 in our study. With regard to the next pregnancy, the risk of chromosomal abnormalities was primarily associated with advanced age rather than PKS pregnancy history.

Our study has several limitations. It has a small sample size and FISH validation was not performed on all samples to determine the abnormal cell composition.

In conclusion, our study showed the atypical manifestations of PKS in prenatal diagnosis such as ultrasound finding and diagnostic specimens. PKS may occur even if the ultrasound of the second trimester is normal. Advanced parental age is the most important factor for PKS. Only by a combination of cytogenetics and molecular techniques can an accurate diagnosis of PKS be made.

## CONFLICT OF INTEREST

The authors declare they have no conflict of interest.

## AUTHOR CONTRIBUTION

**Xiaoqing Wu:** Data curation (equal); Formal analysis (equal); Writing‐original draft (lead). **Xiaorui Xie:** Formal analysis (equal); Investigation (equal). **Linjuan Su:** Data curation (equal). **Na Lin:** Formal analysis (equal); Investigation (equal). **Bin Liang:** Data curation (equal); Formal analysis (equal); Investigation (equal). **Nan Guo:** Formal analysis (equal). **Qingquan Chen:** Software (equal). **liangpu xu:** Project administration (equal); Writing‐review & editing (equal). **Hailong Huang:** Data curation (equal); Resources (equal).

## Data Availability

All data supporting the findings of this study are available within the article.
